# Salvage robotic-assisted subtotal esophagectomy after chemoradiotherapy for unresectable locally advanced esophageal sarcoma: A case report

**DOI:** 10.3892/ol.2026.15706

**Published:** 2026-06-16

**Authors:** Masaaki Yamamoto, Atsushi Takeno, Kiyoshi Mori, Yumiko Hirose, Shinji Tokuyama, Yuki Matsui, Reishi Toshiyama, Kenji Kawai, Yusuke Takahashi, Kenji Sakai, Naoki Hama, Takeshi Kato, Koji Takami, Motohiro Hirao

**Affiliations:** 1 Department of Surgery, National Hospital Organization Osaka National Hospital, Osaka 540-0006, Japan; 2 Department of Central Laboratory and Surgical Pathology, NHO Osaka National Hospital, Osaka 540-0006, Japan

**Keywords:** esophagus, sarcoma, carcinosarcoma, robot, esophagectomy, salvage, CRT

## Abstract

Esophageal sarcoma is an extremely rare tumor, and a recommended treatment strategy remains unestablished. The present report describes, to the best of our knowledge, the first reported case of unresectable esophageal sarcoma treated with chemoradiotherapy (CRT) followed by salvage robot-assisted minimally invasive esophagectomy (RAMIE). An 82-year-old man presented with dysphagia for solid foods. An esophagogastroduodenoscopy revealed an elevated tumor in the middle thoracic esophagus at a distance of 30–35 cm from the incisors, with preserved endoscopic passage and no additional lesions in the stomach or duodenum. A biopsy demonstrated sarcoma. Enhanced computed tomography showed a middle thoracic esophageal mass, with bulky mediastinal lymph nodes (nos. 101R and 106recR) invading the trachea. No distant metastases were detected, and tumor marker levels remained within normal limits. The patient was diagnosed with esophageal sarcoma with mediastinal lymph node metastases. As the metastatic nodes demonstrated airway invasion, definitive CRT (dCRT) was selected. The regimen consisted of radiotherapy (50.4 Gy in 28 fractions) and fluorouracil plus cisplatin. Following dCRT, the tumor and lymph nodes decreased in size, and a partial response was achieved, making the tumor resectable. Salvage RAMIE with two-field lymphadenectomy, including cervical lymph node dissection of stations 101R and 101L and upper mediastinal dissection, including station 106recR, was subsequently performed, followed by gastric tube reconstruction via the posterior mediastinal route. Pathological examination of the resected specimen revealed no residual tumor. Although a therapeutic effect was observed, a limitation remains in that a definitive diagnosis could not be fully established. A total of 14 months have passed since the initiation of treatment, with no evidence of recurrence. In conclusion, this patient with unresectable locally advanced esophageal sarcoma achieved a complete pathological response after multidisciplinary treatment, with no recurrence observed during the short-term follow-up period.

## Introduction

Esophageal sarcoma is an extremely rare malignant tumor, accounting for <1% of all esophageal malignancies (1–3). In contrast to the predominance of squamous cell carcinoma (SCC) and adenocarcinoma in esophageal cancer, primary mesenchymal tumors of the esophagus are uncommon, and their clinicopathological characteristics and optimal treatment strategies remain poorly defined (1,2). Due to this rarity, evidence regarding prognosis and standard management is limited to small case series and case reports.

Esophageal sarcoma may arise as a pure sarcoma or coexist with epithelial malignant components, particularly SCC, which is referred to as carcinosarcoma (1,3). Histologically, these tumors often demonstrate spindle-cell proliferation and may exhibit heterogeneous differentiation patterns, making accurate pathological diagnosis challenging, especially in small biopsy specimens (2,3). Clinically, patients commonly present with dysphagia, weight loss, chest discomfort or bleeding, similar to the symptoms of conventional esophageal carcinoma (1,2). Compared with ordinary SCC, esophageal sarcoma tends to form polypoid or protruding intraluminal masses and may grow rapidly despite relatively preserved swallowing function during the early phase (2,3).

Although surgery remains the mainstay of treatment for resectable disease, no consensus has been established regarding perioperative chemotherapy or chemoradiotherapy (CRT) due to the rarity of this tumor (1–3). In unresectable locally advanced cases, treatment strategies are generally adapted from those for esophageal SCC. Several reports have suggested that esophageal sarcoma may respond favorably to CRT, including cases with invasion into adjacent organs (4,5). However, evidence regarding salvage surgery after definitive CRT (dCRT) for esophageal sarcoma is extremely limited.

Robot-assisted minimally invasive esophagectomy has been increasingly adopted for locally advanced esophageal cancer due to its superior visualization and precise mediastinal dissection capabilities, particularly in technically demanding salvage settings after CRT. Nevertheless, to the best of our knowledge, no previous report has described salvage robot-assisted subtotal esophagectomy after dCRT for unresectable esophageal sarcoma with tracheal invasion.

The present study reports a rare case of unresectable locally advanced esophageal sarcoma with bulky mediastinal lymph node metastases invading the trachea, which achieved a pathological complete response after multidisciplinary treatment consisting of dCRT followed by salvage robot-assisted minimally invasive esophagectomy.

## Case report

### Patient

**Figure 1.**
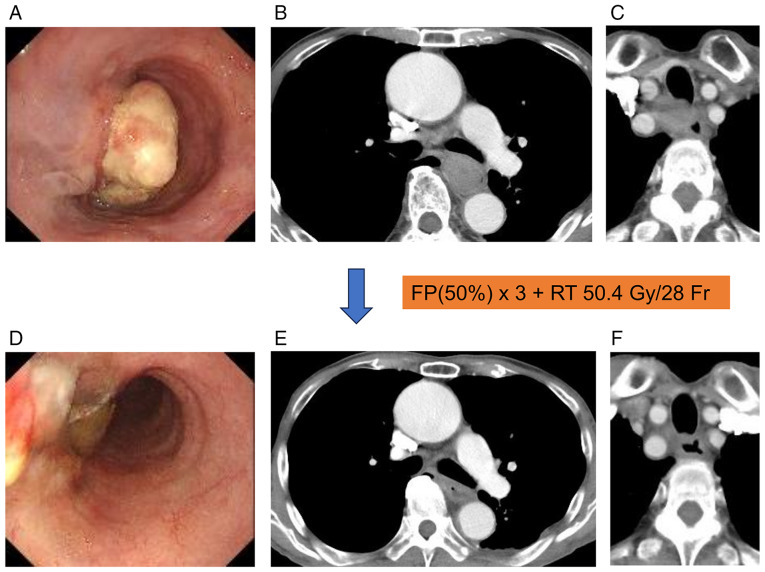
Findings of the EGD and CT scans before and after CRT. (A) EGD revealed an elevated tumor in the esophagus at a distance of 30–35 cm from the incisors. (B) Enhanced CT revealed a tumor located in the middle thoracic esophagus with invasion of the left main bronchus. (C) Enhanced CT revealed bulky lymph nodes (nos. 101R and 106recR) invading the trachea. (D) After CRT, EGD revealed shrinkage of the primary esophageal tumor. (E) Post-CRT enhanced CT demonstrated reduction of the primary esophageal tumor. (F) Post-CRT enhanced CT demonstrated shrinkage of the metastatic lymph nodes. FP, 5-fluorouracil + cisplatin; RT, radiation; Fr, fraction; EGD, esophagogastroduodenoscopy; CT, computed tomography; CRT, chemoradiotherapy.

**Figure 2.**
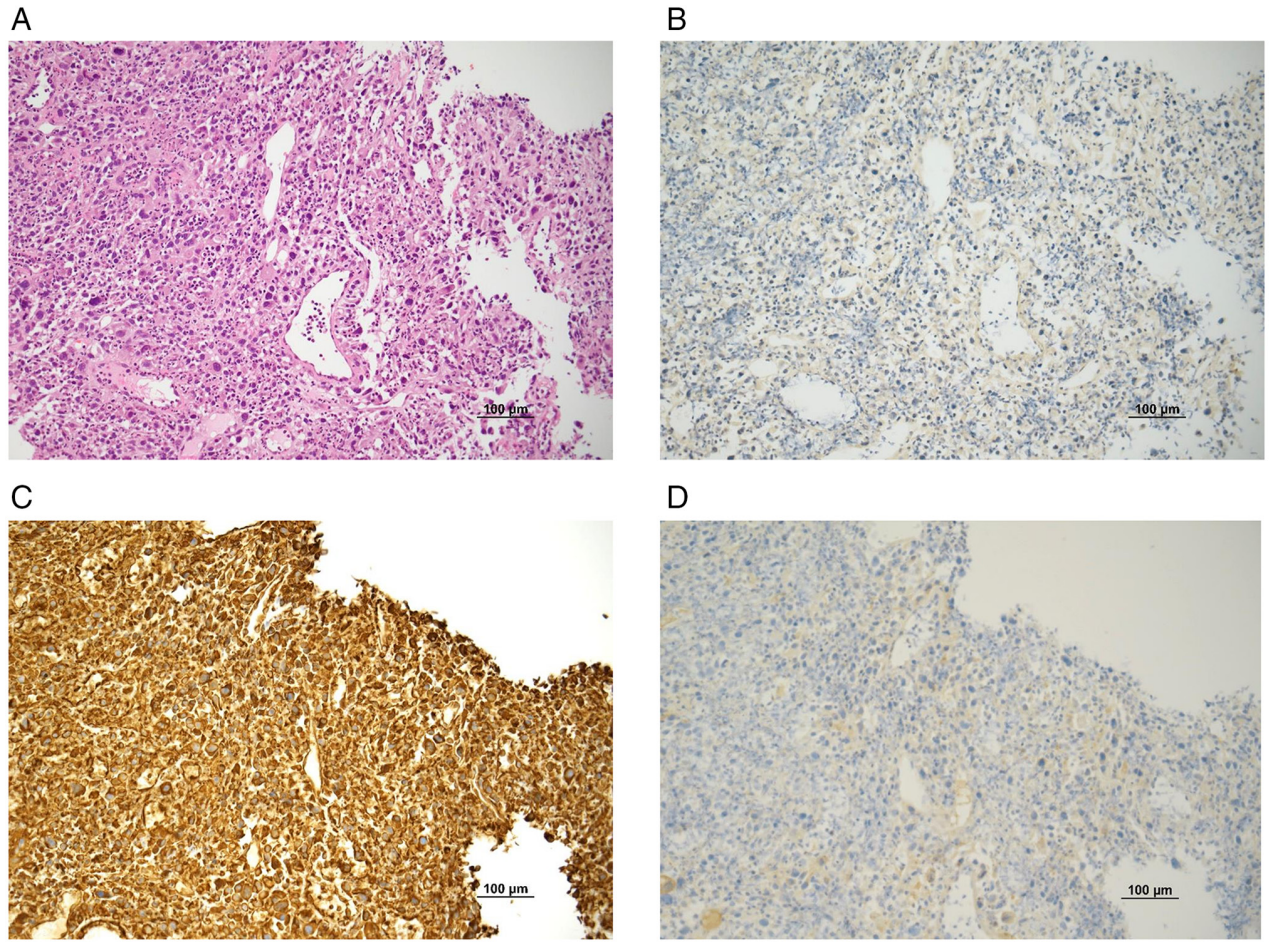
Pathological biopsy of an esophageal tumor at a magnification of ×20 (scale bar, 100 µm). (A) Hematoxylin and eosin staining showing solid proliferation of atypical epithelioid cells with enlarged, oval to irregular nuclei. (B) AE1/AE3 immunostaining is negative. (C) Vimentin immunostaining showing strong diffuse positivity. (D) Melan-A immunostaining is negative.

An 82-year-old man presented to Shitennoji hospital (Osaka, Japan) in October 2024 with dysphagia for solid food. The medical history included rectal cancer surgery, a thoracic aortic aneurysm and aortic valve regurgitation. The patient received a diagnosis of an esophageal tumor and was referred to National Hospital Organization Osaka National Hospital (Osaka, Japan). An esophagogastroduodenoscopy (EGD) revealed an elevated tumor in the esophagus at a distance of 30–35 cm from the incisors (Fig. 1A). Endoscopic passage was possible, and no additional tumors were detected in the stomach or duodenum. Contrast-enhanced computed tomography (CT) revealed a tumor in the mid-thoracic esophagus without evidence of distant metastasis, accompanied by enlarged lymph nodes (nos. 101R and 106recR) compressing the trachea (Fig. 1B and C). CT further showed that the tumor was adherent to approximately one-third of the tracheal circumference, including the entire tracheal membranous portion, over a length of 5 cm along the esophageal axis. On axial imaging, the angle of tracheal involvement from the tumor center was 70°. Tumor compression resulted in deformation of the tracheal cartilage rings and the membranous portion, with the membranous wall clearly protruding into the tracheal lumen. Based on these findings, tracheal invasion by the enlarged lymph nodes was suspected. A bronchoscopy was proposed for further investigation; however, the patient did not consent. Carcinoembryonic antigen and SCC antigen levels remained within the normal ranges. Histopathological examination of the endoscopic biopsy revealed a solid proliferation of atypical epithelioid cells with enlarged, oval to irregular nuclei on hematoxylin and eosin staining. During immunohistochemical analysis (Data S1), AE1/AE3 immunostaining was negative, while Vimentin immunostaining demonstrated strong diffuse positivity, indicating the absence of an epithelial component. Melan-A immunostaining was also negative. Based on these findings, the endoscopic biopsy was diagnosed as sarcoma (Fig. 2A-D). According to the aforementioned findings and the 8th edition of the Union for International Cancer Control Tumor-Node-Metastasis classification, the patient was diagnosed with locally advanced esophageal sarcoma, cT4 (101R-106recR-trachea) N1M0, cStage IVA, accompanied by enlarged lymph nodes (nos. 101R and 106recR) infiltrating the trachea (6,7). After informed consent was obtained from the patient, definitive CRT was selected as the bulky lymph nodes (nos. 101R and 106recR) had invaded the trachea and may cause immediate airway obstruction.

The treatment regimen consisted of RT (50.4 Gy in 28 fractions, 1.8 Gy/day, 5 days per week) combined with 5-fluorouracil + cisplatin (FP) at doses of 560 mg/m^2^ intravenous 5-fluorouracil on days 1–4 and 56 mg/m^2^ intravenous cisplatin on day 1. The doses of 5-fluorouracil and cisplatin were reduced by 50% compared to the recommended dosage for esophageal cancer (SCC) due to advanced age and impaired renal function.

After RT (50.4 Gy) and three cycles of FP therapy, EGD and CT demonstrated shrinkage of the primary tumor and lymph nodes. One additional cycle of FP therapy was then performed. Finally, a partial response (PR) was determined according to the Response Evaluation Criteria in Solid Tumors, version 1.1 (8), and the tumor was considered resectable based on findings from EGD and CT after RT (50.4 Gy) with three cycles of FP therapy (Fig. 1D-F). The patient declined additional examinations, including positron emission tomography. During CRT, the patient experienced grade 2 hypokalemia and grade 1 diarrhea according to the Common Terminology Criteria for Adverse Events, version 5.0 (9).

As the EGD and CT scans suggested the possibility of residual tumors, a discussion was conducted with the patient with regard to whether to continue chemotherapy or undergo surgery. After informed consent was obtained again, robot-assisted minimally invasive esophagectomy (RAMIE) with two-field lymphadenectomy plus no. 101R and no. 101L node dissection and gastric tube reconstruction via a posterior mediastinal route were performed as salvage surgery following CRT.

### Operative technique

**Figure 3.**
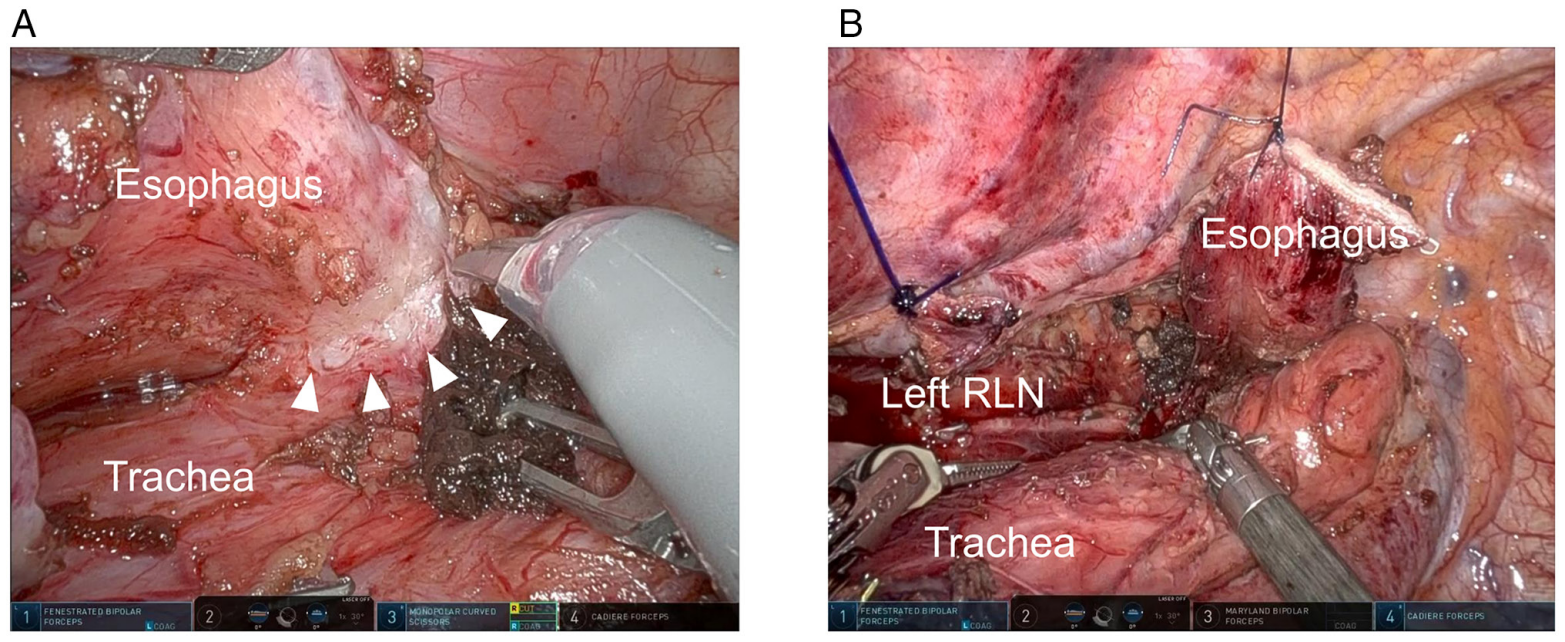
Intraoperative view of the intrathoracic region. (A) Fibrotic changes after chemoradiotherapy were observed in the primary esophageal tumor around the paraesophageal lymph nodes and trachea (white arrowheads). (B) After completing a dissection of the fibrotic tissue, the thoracic procedure was concluded. RLN, recurrent laryngeal nerve.

The da Vinci Xi Surgical System (version 4) (Intuitive Surgical Operations, Inc.) was used during the thoracic phase of the surgery. Under general anesthesia, the patient was placed in the prone position.

da Vinci ports (8 mm) were inserted as follows: The 1st arm port in the 10th intercostal space (ICS) a long a line parallel to the anterior axillary line (AL) passing through the inferior angle of the scapula; the 2nd arm port in the 8th ICS on the middle AL; the 3rd arm port in the 6th ICS on the middle AL; and the 4th arm port in the 4th ICS on the anterior AL. Assistant ports included a 5-mm port in the 9th ICS on the anterior AL and a 12-mm port in the 7th ICS on the anterior AL. Fibrotic changes after CRT were observed in the primary esophageal tumor around the paraesophageal lymph nodes and trachea (Fig. 3A). After completing dissection of the fibrotic tissue, the thoracic procedure was concluded (Fig. 3B).

After completing the thoracic phase, the patient was repositioned in a 5 head-up tilt. The abdominal and cervical phases were performed simultaneously. During the abdominal phase, an upper abdominal lymphadenectomy and gastric tube construction were performed laparoscopically. The first port (12 mm) for the camera was placed at the umbilicus. Four additional ports were placed: A 5-mm port in the right hypochondrium; a 5-mm port in the right upper abdomen between the right hypochondrial port and the umbilicus; a 5-mm port in the left hypochondrium; and a 12-mm port in the left upper abdomen between the left hypochondrial port and the umbilicus. For the cervical phase, a cervical lymph node dissection was performed, and complete removal and resection of the tumor were achieved. The gastric tube was pulled up through a posterior mediastinal route.

### Clinical outcomes

**Figure 4.**
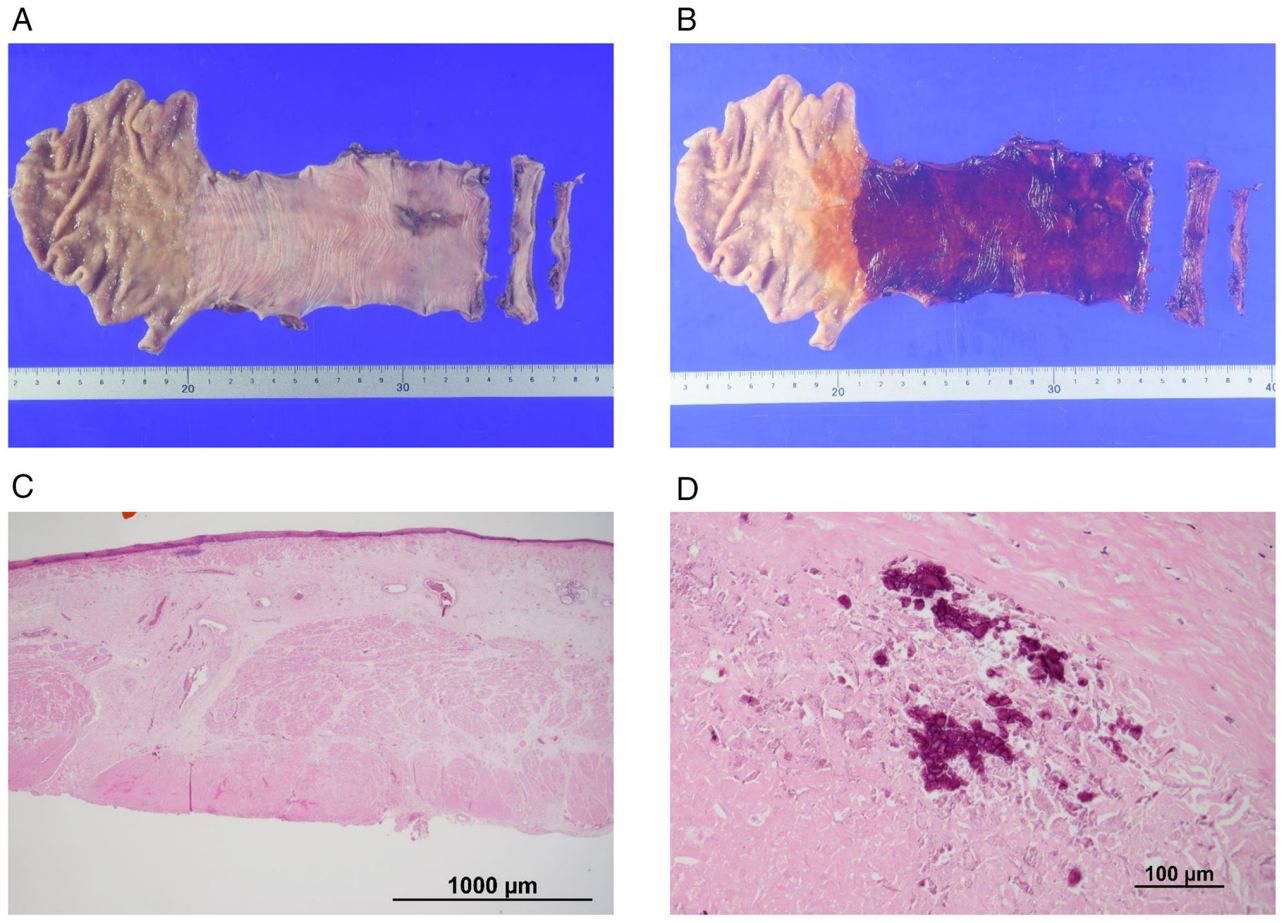
Surgical specimens of the esophagus and stomach. (A) Resected specimen of the esophagus and stomach and (B) Lugol-stained resected specimen. (C) Microscopic images of the esophageal specimen stained with H&E at a magnification of ×4 showing fibrotic changes from the lamina propria to the submucosa, with focal extension into the muscularis propria. Scale bar, 1,000 µm. No residual tumor was identified, including within the intraepithelial component. (D) The H&E-stained no. 101R lymph node at a magnification of ×20 shows fibrotic changes without any residual tumor. Scale bar, 100 µm. H&E, hematoxylin and eosin.

Extubation was performed on postoperative day (POD) 1. However, on POD 3, the patient developed pneumonia and difficulty expectorating sputum; therefore, intravenous ampicillin/sulbactam (UNASYN^®^) at a dose of 3 g three times daily was initiated and continued for 7 days. Reintubation was also required. Further examination by the otolaryngology team did not reveal recurrent laryngeal nerve paralysis. Although oxygenation stabilized, difficulty with sputum expectoration persisted, and a tracheostomy was performed on POD 8. The pneumonia improved by POD 12, and antibiotic administration was discontinued. Mechanical ventilation was discontinued on POD 17. A tracheostomy mask was used on POD 31, and a heat and moisture exchanger (artificial nose) were introduced on POD 45. Subsequently, rehabilitation focused primarily on swallowing training with thickened oral intake, and the patient was discharged on POD 93. Pathological examination of the resected specimen revealed no residual tumor, and the therapeutic effect of CRT was evaluated as grade 3 (no variable cancer cells detected) based on Japanese Classification of Esophageal Cancer, 12th edition (Fig. 4A-D) (7,10). After considering the patient's overall condition, no adjuvant postoperative therapy was administered. A total of 14 months have passed since the initiation of treatment (10 months since surgery), with no evidence of recurrence. The patient has been followed up with tumor marker assessments every 3 months and CT every 6 months.

## Discussion

To the best of our knowledge, the current report presents the first description of a patient with esophageal sarcoma who achieved a grade 3 pathological response following a subtotal esophagectomy RAMIE after CRT (7,10).

Esophageal sarcoma represents a rare malignancy and accounts for 0.5–2.8% of all esophageal cancer cases (1–3). This condition occurs more frequently in men and typically arises in the middle thoracic esophagus (1,2). The macroscopic appearance is often polypoid, and dysphagia due to impaired food passage has been reported as a common presenting symptom (1,2). Esophageal sarcoma frequently coexists with SCC, a presentation referred to as carcinosarcoma (1). When the sarcomatous component predominates, a polypoid morphology is generally observed, whereas carcinomatous dominance more commonly results in an ulcerative lesion (11,12).

Carcinosarcoma is often not difficult to distinguish clinically based on its macroscopic characteristics. However, it is a biphasic tumor composed of both sarcomatous and carcinomatous components. Therefore, depending on the biopsy site, the pathological diagnosis may reflect only one component, potentially reducing the accuracy of the preoperative diagnosis. Furthermore, even when a biopsy specimen is obtained from the sarcomatous portion of the tumor, the irregular morphology of the cells can make differentiation between sarcoma and poorly differentiated carcinoma challenging (13). Although the origin of the sarcomatous component remains unclear, the metaplastic theory, which proposes that the sarcomatous component arises monoclonally from a single ancestral cell, is widely accepted (14,15). Specifically, the sarcomatous component may arise from metaplastic changes during the epithelial-mesenchymal transition of the carcinomatous component. This concept is supported by observations of transition zones between the two components and shared gene mutations, including TP53 mutations and p53 upregulation, in both components (16,17).

Management of esophageal sarcoma generally follows the therapeutic principles applied to esophageal SCC, as frequent coexistence with SCC and a high likelihood of lymph node metastasis have been described (4). Specifically, in resectable cases, surgical resection or surgical resection following neoadjuvant chemotherapy is commonly performed. RT or CRT remains an alternative for patients with unresectable tumors or for those unable to tolerate surgery. In Japan, concurrent CRT consisting of 5-fluorouracil and cisplatin with RT has been widely adopted as a standard treatment for unresectable locally advanced esophageal cancer, as demonstrated in the Japan Clinical Oncology Group 9516 study (18). Based on these criteria, CRT was selected for the present patient due to unresectability at diagnosis and potential airway obstruction. Following treatment, the tumor demonstrated a marked reduction in size and was subsequently deemed resectable.

Table I summarizes the results of CRT for carcinosarcoma (4,5,12,19–22). Among the seven cases diagnosed with carcinosarcoma before treatment, only two cases were diagnosed as SCC alone in the pathological examination after surgical resection following CRT, with no sarcomatous component detected. Both cases exhibited a PR, suggesting that CRT was effective against the sarcomatous component in 28.6% (2/7) of cases (19,20). In the remaining five cases, the pathological diagnosis remained carcinosarcoma both before and after CRT, with three cases showing PR (60%, 3/5) and two cases (40%, 2/5) being indeterminate (12,20,21). Furthermore, among the two cases diagnosed as SCC before treatment, postoperative pathological examination after CRT revealed carcinosarcoma in one case and sarcoma alone in the other (5,19,22). These findings suggest that preoperative endoscopic biopsies may have sampled only the SCC component, indicating that an accurate diagnosis of carcinosarcoma may be difficult depending on the site of the preoperative endoscopic biopsy. Furthermore, considering sensitivity to CRT, the SCC component appears to be more responsive than the sarcomatous component, which may explain why only the sarcomatous component remained after CRT in some cases (19,21,22). Regarding RT alone for carcinosarcoma, there are reports of a PR in patients with unresectable carcinosarcoma who received RT alone, although differences in radiosensitivity between the sarcomatous and carcinomatous components remain unclear (19,23,24). While long-term prognosis remains uncertain, temporary local control may be achieved with RT. Chemotherapy alone for carcinosarcoma appears to demonstrate greater efficacy against the carcinomatous component than against the sarcomatous component, whereas the therapeutic effect on the sarcomatous component appears limited (19,21,22).

Regarding surgery, surgical procedures following the pattern of esophageal SCC, such as transthoracic subtotal esophagectomy with lymph node dissection, are frequently performed (3). Recent years have shown widespread adoption of a minimally invasive esophagectomy, with thoracoscopic esophageal procedures being actively utilized. Moreover, the use of RAMIE has expanded rapidly in clinical practice (25,26). In the present case, RAMIE was performed as salvage treatment for a patient with esophageal sarcoma. Robotic surgery provides multiple advantages for esophagectomy compared with thoracoscopic or open approaches (25–27). RAMIE enables highly precise surgical procedures through three-dimensional magnified visualization, tremor elimination and multiple articulations of the endo-wrist. This technique appears particularly beneficial for post-CRT patients with esophageal sarcoma undergoing salvage surgery, where these technical advantages can be fully utilized.

In the present case, the pre-treatment diagnosis was, strictly speaking, sarcoma. As the resected specimen showed no residual tumor, a definitive conclusion could not be drawn. Therefore, this remains a limitation of the present report. However, considering the reported frequency of esophageal sarcoma, we infer that the pre-treatment biopsy likely sampled the sarcomatous component of a carcinosarcoma (3). Several reports have reported the use of CRT for patients with esophageal carcinosarcoma (Table I) (4,5,12,19–22). The compiled cases highlight several important observations regarding esophageal sarcoma and carcinosarcoma treated with esophagectomy following CRT. First, a clear discrepancy has emerged between pre-treatment biopsy diagnosis and the final pathological diagnosis. In multiple cases, biopsies suggested SCC or carcinosarcoma, although post-surgical histopathology revealed either sarcomatous or carcinomatous components alone. This finding emphasizes the diagnostic challenge in accurately identifying esophageal sarcoma preoperatively, likely due to tumor heterogeneity and the limited sampling inherent to endoscopic biopsy. Second, the clinical response to CRT was generally favorable in most cases, with a PR observed even in tumors initially classified as T3 or T4. This suggests that esophageal sarcoma, traditionally considered less responsive to CRT, may exhibit substantial sensitivity under certain conditions, supporting the potential role of CRT as a neoadjuvant strategy. Notably, in the current case, the patient achieved grade 3 tumor regression after CRT, which represents the highest response observed in this series (7,10). The present case demonstrates that curative outcomes can be achieved with CRT even when the tumor is deemed unresectable at initial diagnosis, suggesting that CRT alone may represent a highly effective treatment option. Finally, these findings collectively highlight the need for treatment strategies aligned with esophageal SCC protocols when sarcoma is suspected or diagnosed via limited biopsy, due to both diagnostic uncertainty and the potential for a notable response to CRT. In other words, even if a patient presents with a carcinosarcoma or sarcoma, they may be candidates for multimodality treatment.

In conclusion, the present study reports a case in which CRT was performed followed by robotic-assisted subtotal esophagectomy for unresectable esophageal sarcoma with infiltration into other organs. The patient achieved a pathological complete response after multimodality treatment and remains disease-free at 14 months after treatment initiation (10 months after surgery).

## Supplementary Material

Supporting Data

## Figures and Tables

**Table I.** Cases of esophageal carcinosarcoma treated with preoperative chemoradiotherapy followed by esophagectomy.

First author, year	Age, years	Sex	Biopsy pathological diagnosis	Location	TNM before treatment	Chemotherapy	Radiation, Gy	Clinical response	TNM after surgery	Histological response^a^	Pathological diagnosis	OS time, months	Dead/alive	(Refs.)
Okuda *et al*, 2005	57	M	SCC	Mt	T3N2M1	FP	30	SD	T4N2M1	N/A	Carcinosarcoma	4	Dead	(5)
Zuiki *et al*, 2009	50	M	Carcinosarcoma	UtCe	T3N1M0	FP	40	PR	T1bN2M0	N/A	SCC	35	Alive	(19)
	66	M	SCC	Lt	N/A	FP	40.8	PR	T1bN0M0	N/A	Sarcoma	19	Alive	
Kuo (4) *et al*, 2010	68	M	Carcinosarcoma	Ut	T3N1M0	N/A	N/A	N/A	N/A	N/A	N/A	27	Alive	(4)
	45	M	Carcinosarcoma	Lt	T4N1M0	N/A	N/A	N/A	N/A	N/A	N/A	6	Alive	
Kobayashi *et al*, 2010	68	M	Carcinosarcoma	CeUt	T3N1M0	S1 + CDDP	40	PR	TisN0M0	Grade 2	SCC	60	Alive	(20)
	64	M	Carcinosarcoma	Ce	T2N1M0	FP	38	PR	T1aN0M0	Grade 2	Carcinosarcoma	7	Dead	
Katsuya *et al*, 2017	67	F	Carcinosarcoma	Mt	T1bN1M0	FP	50.4	PR	T1bN0M0	Grade 1	Carcinosarcoma	10.9	Dead	(21)
	73	F	Carcinosarcoma	Lt	T2N1M0	FP	41.4	PR	T1bN0M0	Grade 2	Carcinosarcoma	47	Alive	
Yamauchi *et al*, 2022	65	M	SCC	N/A	T3N1M0	FP	60	N/A	T2N0M1	N/A	Carcinosarcoma	12	Dead	(22)
Yang *et al*, 2022	70	M	Carcinosarcoma	N/A	T3N1M0	S1	44.94	PR	T2N0M0	Grade 1	Carcinosarcoma	37.4	Alive	(12)
	62	M	Carcinosarcoma	N/A	T1N1M0	EP	47.08	PR	T3N0M0	N/A	Carcinosarcoma	22.7	Alive	
Present case	81	M	Sarcoma	MtLt	T4N1M0	FP	50.4	PR	T0N0M0	Grade 3	Sarcoma	14	Alive	

a Japanese Classification of Esophageal Cancer, 12th edition; Ce, cervical esophagus; Ut, upper thoracic esophagus; Mt, middle thoracic esophagus; Lt, lower thoracic esophagus according to the Japanese Classification of Esophageal Cancer, 12th edition; TNM, Tumor-Node-Metastasis classification according to the Japanese Classification of Esophageal Cancer, 12th edition; SCC, squamous cell carcinoma; FP, 5-fluorouracil + cisplatin; EP, etoposide + cisplatin; CDDP, cisplatin; S1, tegafur-gimeracil-oteracil potassium; SD, stable disease; PR, partial response; OS, overall survival.

## Data Availability

The data generated in the present study are included in the figures and/or tables of this article.

## Abbreviations

EGD: esophagogastroduodenoscopy
CT: computed tomography
SCC: squamous cell carcinoma
CRT: chemoradiotherapy
dCRT: definitive chemoradiotherapy
FP: 5-fluorouracil + cisplatin
RAMIE: robot-assisted minimally invasive esophagectomy
ICS: intercostal space
AL: axillary line
POD: postoperative day
