# Supplementary material for: Salvage robotic-assisted subtotal esophagectomy after chemoradiotherapy for unresectable locally advanced esophageal sarcoma: A case report
Source: Oncol Lett. 2026 Jun 16;32(2):351. doi: 10.3892/ol.2026.15706 (PMC13311904; doi:10.3892/ol.2026.15706)
Supplement: Supporting Data [file Supplementary_Data.pdf]

Data S1

Histopathological examination. Formalin-fixed (10% neutral buffered formalin, room temperature; 24 h for biopsy specimens and 9 h for surgical specimens), paraffin-embedded tissue blocks were sectioned at a 1.25-μm (biopsy specimens) and 1.5-μm (surgical specimens) thickness and mounted on silane-coated glass slides. For macroscopic examination prior to tissue sampling of surgical specimens, undiluted Lugol's iodine solution was applied topically to the entire mucosal surface at room temperature for 3 min to identify iodine-unstained areas. Sections were stained with hematoxylin and eosin (H&E) using VENTANA HE 600 Hematoxylin (cat. no. 07024282001) and VENTANA HE 600 Eosin (cat. no. 06544304001) reagents according to the manufacturer's default protocol (hematoxylin, 2 min; differentiating solution, 0.5 min; eosin, 2 min; system operating temperature, 15-32˚C). All histological sections were examined by light microscopy (BX53; Olympus Corporation).

Immunohistochemical (IHC) staining. Formalin-fixed (10% neutral buffered formalin, room temperature, 9 h), paraffin-embedded tissue blocks were sectioned at a 4-μm thickness and mounted on silane-coated glass slides. Sections were baked at 60˚C for 30 min before IHC staining. All IHC procedures were performed using an automated slide staining system, BenchMark ULTRA (Roche Tissue Diagnostics).

Unless otherwise specified, the following instrument-specific reagents were used for all stains: EZ Prep solution (10X; deparaffinization buffer; catalog no. 950-102), Reaction Buffer (Tris-based buffer, pH 7.6-7.8; catalog no. 950-300), Cell Conditioning 1 (CC1; Tris-EDTA-based antigen retrieval buffer, pH 7.8; catalog no. 950-124), ultraView Universal DAB Detection Kit (biotin-free HRP multimer-based detection system; catalog no. 760-500) and Hematoxylin II (catalog no. 790-2208) (all Roche Tissue Diagnostics).

The primary antibodies used were as follows: Mouse monoclonal anti-pan-cytokeratin antibody (clone AE1/AE3/PCK26; catalog no. 760-2595; ready-to-use), mouse monoclonal anti-Melan A/MART-1 antibody (clone A103; catalog no. 790-2990; ready-to-use) and mouse monoclonal anti-vimentin antibody (clone V9; catalog no. 790-2917; ready-to-use) (all Roche Tissue Diagnostics).

Appropriate external positive control tissues and negative reagent controls were included in each staining run.

Automated solvent-free deparaffinization was performed using EZ Prep solution at 75˚C according to the standard BenchMark ULTRA protocol. Heat-induced epitope retrieval was carried out with CC1 at 95˚C. Retrieval conditions were adjusted for each antibody as follows: Pan-cytokeratin AE1/AE3, CC1 treatment for 36 min followed by Protease 1 treatment for 4 min at room temperature; Melan A/MART-1 (A103), CC1 treatment for 64 min; and vimentin (V9), CC1 treatment for 32 min, according to the manufacturer's recommendations and internal validation procedures.

Endogenous peroxidase activity was blocked using the peroxidase inhibitor included in the ultraView Universal DAB Detection Kit and automatically applied according to the manufacturer's protocol. Ready-to-use primary antibodies were incubated at 36˚C for 8 min (pan-cytokeratin AE1/AE3), 16 min (Melan A/MART-1) and 32 min (vimentin). Incubation times were determined based on the manufacturer's instructions and internal validation data.

Signal detection was performed using the ultraView Universal DAB Detection Kit according to the manufacturer's protocol, including incubation with HRP-labeled multimer for 8 min and DAB chromogen for 8 min. No additional signal amplification procedures were used.

Slides were counterstained with Hematoxylin II for ~8 min, followed by bluing reagent for 4-8 min. Slides were subsequently rinsed in tap water, dehydrated through graded ethanol solutions, cleared in xylene, and coverslipped using a synthetic mounting medium.
